# Modification of Ti13Nb13Zr Alloy Surface via Plasma Electrolytic Oxidation and Silver Nanoparticles Decorating

**DOI:** 10.3390/ma18020349

**Published:** 2025-01-14

**Authors:** Przemysław Gołasz, Agnieszka Płoska, Viktoriia Korniienko, Kateryna Diedkova, Yuliia Varava, Rafał Zieliński, Maksym Pogorielov, Wojciech Simka

**Affiliations:** 1 Department of Inorganic Chemistry, Analytical Chemistry, and Electrochemistry, Faculty of Chemistry, Silesian University of Technology, 44-100 Gliwice, Poland; pg292060@student.polsl.pl (P.G.); agniplo838@student.polsl.pl (A.P.); m.pogorielov@gmail.com (M.P.); 2 Chemistry Students Research Society, Faculty of Chemistry, Silesian University of Technology, 44-100 Gliwice, Poland; 3 Institute of Atomic Physics and Spectroscopy, University of Latvia, Jelgavas iela 3, LV-1004 Riga, Latvia; v.kornienko@med.sumdu.edu.ua (V.K.); kateryna.diedkova@lu.lv (K.D.); 4 Biomedical Research Centre, Sumy State University, R-Korsakova Street, 40007 Sumy, Ukraine; yuliia.varava@gmail.com; 5 Stomatologia na Księżym Młynie, 90-365 Łodz, Poland; bkost@op.pl

**Keywords:** titanium alloy, silver nanoparticles, implant, surface treatment

## Abstract

The dynamically developing field of implantology requires researchers to search for new materials and solutions. In this study, TiNbZr samples were investigated as an alternative for popular, but potentially hazardous TiAl6V4. Samples were etched, sandblasted, subjected to PEO, and covered in AgNP suspension. Simultaneously, SEM images were taken, and the wettability and roughness of the surface were measured. Samples covered in AgNPs were subjected to biological trials. A six-day measurement of human fibroblast proliferation was conducted to assess biocompatibility, and the population of *E. coli* and *S. aureus* was measured over eight hours. Results showed that the TiNbZr PEO surface is biocompatible with human fibroblast cells and promotes growth. However, deposited AgNPs exhibited only slight effectiveness in decreasing bacterial growth over the first two hours. The results suggest that the method of surface preparation is sufficient and might promote osseointegration. On the other hand, more efficient and reliable methods of application of AgNPs should be researched

## 1. Introduction

The medical industry values titanium and its alloys for their biocompatibility, strength, corrosion resistance, and ability to form a protective TiO_2_ oxide layer on an implant. These qualities make titanium implants suitable for use in almost all fields of surgery, ranging from dentistry and plastics to trauma and cardiothoracic interventions. Titanium alloys’ Young modulus resembles that of a bone, which makes it an excellent bone replacement material. Many titanium alloys are known for clinical applications, but one of the most commonly used is Ti6Al4V. It exhibits exceptional mechanical strength and high similarity in compression and tension to bones [1,2]. However, it has been demonstrated that the impact of the components of this alloy is unfavorable to human health. Vanadium ions exhibit cytotoxic effects and possibly cause type IV allergies [3,4], and aluminum ions presence might cause bone mineralization issues and were linked to the development of Alzheimer’s disease [4,5]. To research alternatives, the Ti13Nb13Zr alloy was chosen as the investigated material in this study. Studies show that niobium does not exhibit cytotoxic effects on human cells [6]. Its alloys with titanium increase the proliferation of osteoblast cells and exhibit superior cellular response of preosteoclasts compared to vanadium alloys with titanium [7,8]. Zirconium is a well-researched biocompatible metal often used in dentistry as zirconia (zirconium(IV) oxide ZrO_2_ [9,10,11]). H. Michelle Grandin’s study shows that binary titanium–zirconium alloys achieve greater osseointegration and biocompatibility compared to pure titanium [12]. The alloy itself was shown to promote the adhesion of human mesenchymal stem cells (MSCs) to the implant surface rather than Ti6Al4V [13].

One of the challenges in implantology is preventing bacterial infections at the implant site. It has been proven that after the successful implantation, the surface of the implant may be covered in a thin, bacterial film, potentially causing infections or inhibiting osseointegration of the implant [14,15,16,17]. Those biofilms are proven to be highly resistant to antibiotics [18] and are responsible for in situ infections and also bloodstream infections [19,20,21,22]. The resistance to conventional antibiotics has led to extensive research on new methods of preventing the formation of these biofilms. One promising approach is coating implants with bacteriostatic or bactericidal coatings. Essentially, such coatings can be divided into three types—anti-adhesive, contact-killing, and releasing types [23].

Anti-adhesive coatings prevent bacteria from adhering and covering the surface of medical devices with bacterial biofilm [24]. Attachment of the macromolecules to the surface of the implant allows them to alter their physical properties [25,26,27,28,29,30], and effectively change conditions of the surface of the implant to be hostile toward the bacteria. In essence, those coatings can be described as hydrophilic and hydrophobic. Hydrophilic anti-adhesive coatings prevent bacterial growth by inhibiting bacterial cell adhesion [31], as bacterial cell walls are lipid-based structures that are hydrophobic. Therefore, they are physically repelled by hydrated surfaces. Some commonly used hydrophilic coatings are hyaluronic acid (HA) [32], sodium alginate [33], PEG and its derivatives [34,35], and polyhydroxypropyl methacrylate (PHPMA) [36] to name just a few. However, their mechanism of action was proven to prevent bone cells from migrating and attaching to implants for exactly those same reasons [37] that repel bacteria. On the other hand, hydrophobic surfaces rely on a self-cleaning mechanism, as body fluids wash their surface thoroughly, rinsing off any bacteria attached. Some of the polymers used as hydrophobic antiadhesive coatings are polydimethylsiloxane [38] and fluorinated polymers [39,40,41]. Also, diamond functional [42] and super anti-wetting nano coatings [43] were developed.

Releasing-type and contact bactericidal coatings are similar in some sense, and they often require special surface preparation to immobilize active particles on the implant. Contact bactericidal coatings contain natural or synthetic particles that are physically or chemically attracted to the surface of the implant. Those particles directly attack the cell membranes of bacteria, not only preventing their adhesion but also killing them [44,45,46,47]. Often used antibacterial particles include chitosan [48,49], antimicrobial peptides (AMPSs) [47,50,51], quaternary ammonium compounds (QACs) [52,53], and inorganic bactericidal nanoparticles [54].

Releasing-type antibacterial coatings essentially work as sponges. Antibiotics, anti-inflammatory or antibacterial drugs, or bactericidal molecules are immobilized in highly developed coatings that release them via diffusion, dissolution, or degradation of certain molecules. The coating itself is a carrier that releases particles on the implantation site, preferably on the first day after surgery, as they are most critical in terms of the formation of bacterial biofilm [55,56,57,58]. Widely used coatings consist of polymers [59,60] and inorganic and organic cements [61]. Also, ceramic layers of oxidized metallic material can introduce desired particles to the surface of the implant. Plasma electrolytic oxidation (PEO) is a promising method of surface development. Not only have PEO coatings been described to increase corrosion resistance and hardness of the implant, but they also increase biocompatibility and promote osseointegration [62,63,64,65,66]. Such coatings are highly porous, allowing drugs to migrate out over a fixed amount of time, which is characteristic of the structure of the surface. Therefore, PEO is a simple, yet effective way of preparing implant surfaces, as only one treatment increases bone-implant integration and stability, and it also forms a releasing-type coating. It is possible to introduce antibiotics into those pores [67]; however, such infusion comes with a risk of antibiotic-resistant bacteria development around the implantation site [68,69]. Among various alternative options, metallic ions are a simple and effective source of active particles in releasing-type coatings. They are thermally physically and chemically resistant, and no known bacterial resistance mechanism has been yet discovered. Various ion sources were extensively researched. However, one of the most prominent ones is silver nanoparticles (AgNP). Their incorporation into the coating may effectively inhibit the growth of both Gram-negative Escherichia coli and Gram-positive Staphylococcus aureus, reducing the risk of implant-associated infections [70,71]. Moreover, the use of silver nanoparticles as a coating material offers a very significant advantage over preventive antibiotic therapy after surgery or using implants with immobilized antibiotics and antibacterial drugs on the surface. Silver nanoparticles have been proven to exhibit strong antimicrobial activity against a broad spectrum of bacteria, fungi, and viruses. Second, they have a low tendency to develop resistance, making them effective in the long term [71]. However, it is worth noting that AgNPs are proven to be cytotoxic and are potentially allergenic [72,73]. For the sake of safety, the amount of AgNPs on the implant surface has to be enough to exhibit the bactericidal effects without showing cytotoxicity. In this study, samples of Ti13Nb13Zr were polished, sandblasted, etched in oxalic acid, oxidized in phosphoric acid via plasma electrolytic oxidation, and then coated with silver nanoparticles using silver nanoparticle suspension. A combination of all these surface treatment techniques is in our opinion novel in the literature. Surface images were taken using SEM imaging, roughness and wettability of samples were measured and biological tests were performed to measure the possible cytotoxic effect of the formed silver nanocoating.

## 2. Materials and Methods

### 2.1. Preparation of the Samples

In this study, Ti13Nb13Zr alloy samples in the form of 10 mm diameter and 4 mm height disks were used. The preparation process involved polishing each disc with abrasive SiC paper of 320 and 800 grit size. Subsequently, they were sandblasted using Al_2_O_3_ with an 80-grit size until a uniform and matte surface finish was achieved. Following this, the samples underwent rinsing in distilled water and degreasing in a mixture of distilled water with detergent and isopropanol for three minutes each using an ultrasonic bath.

### 2.2. Processing the Samples

The samples were etched using a solution consisting of 10% (*w*/*w*) oxalic acid in distilled water at a temperature of 100 °C in a water bath for 70 min. Following the etching process, the samples were rinsed in distilled water for 3 min. Subsequently, plasma electrolytic oxidation was carried out in a controlled-temperature electrolyzer with a 1 M H_3_PO_4_ solution at 10 °C. The PEO process was performed in two stages using a DC power supply (Kiksui, Sapporo, Japan). First, the process occurred under galvanostatic conditions of current density (100 mA/cm^2^) at a limiting voltage (250, 275, 300, 325 V). After the limiting potential was reached, the process continued under potentiostatic conditions of the limiting voltage. The voltage of the PEO process for the further investigated samples was determined by analysis of SEM images (Phenom Pro-X, 15 kV) of samples processed at voltages of 250, 275, 300, and 325 V. Based on this analysis, it was determined that further investigations would be carried out under constant voltage of 250 V. The PEO of these samples was performed as mentioned above, with 100 mA/cm^2^ current density in the galvanostatic part, and at 250 V at the potentiostatic part. Finally, after completion of the PEO treatment, distilled water was used to rinse the disks, and they were left to dry.

Silver nanoparticles solution (3 g/L suspended in PVP—Polyvinylpyrrolidone) (NanoWave, Warsaw, Poland) was used to prepare the solutions (0.5, 0.25, and 0.125 g/L) by diluting it with distilled water. Prepared, oxidized, and dried samples were divided into three groups. Then, 20 μL of solutions of the silver nanoparticles were applied accordingly on the samples using the automatic pipette and then were left to dry. The samples labels were shown in Table 1.

### 2.3. Surface Characteristics

The samples were investigated using the Phenom Pro-X with an applied voltage of 15 kV. SEM images were taken, and 3D images were generated for the polished, sandblasted, etched, oxidized, and treated with AgNP suspension samples. The sample’s average arithmetical roughness of a surface (Sa) was measured using the Phenom Pro-X (λs: 200 nm, λc: 198.61 µm). Also, the EDX analysis of the atomic composition of the investigated samples was conducted. Furthermore, the wettability of the samples was determined using a goniometer (DataPhysics, OCA 15EC, Filderstadt, Germany) by measuring contact angle (CA). Then, 0.5 µL of water was dropped on the samples, and the CA between the disk surface and a water droplet was measured. Each sample’s CA was measured five times from the left and right sides of the water droplet, and the mean value of those measurements was calculated. Also, standard deviations of the measured CAs were obtained.

### 2.4. Biocompatibility and Biological Tests

Biocompatibility test of the samples was performed on human dermal fibroblasts (HDF) (Pharmacy program research group, Medical Faculty of the Latvian University) with the 7th passage (P7). The Institute of Experimental and Clinical Medicine Ethics Committee, University of Latvia, approved the use of primary cells from frozen primary cell stock-No. 71-35/17. The materials were sterilized by autoclaving for 15 min at a pressure of 102.6 kPa and a temperature of 121 °C. Dermal fibroblasts were thawed and cultured according to the Sigma-Aldrich protocol [74]. Cells were seeded onto culture flasks at a density of 5000 cells/cm^2^ under standard conditions of 37 °C, 5% CO_2_, with medium changes every 2–3 days. The cell culture medium containing Dulbecco’s modified Eagle’s medium/nutrient mixture F-12 (DMEM/F-12 3:1 *v*/*v*), L-glutamine, antibiotics (penicillin, streptomycin, and amphotericin B), and 10% fetal bovine serum. All cell culture reagents were purchased from Sigma-Aldrich, St. Louis, MO, USA. The sterilized samples were placed in a 24-well plate and cells were seeded on the surface of samples at a density of 10,000 cells/cm^2^. As a positive control, we used wells with cells without samples, and as a negative control, we used wells with medium without cells and samples. The Alamar blue assay was utilized to evaluate cell adhesion and growth. After 24 h, this colorimetric test measured cell adhesion. It also assessed cell proliferation on days 4 and 6. An amount, accounting for 10% of the entire volume, of Alamar blue (procured from Sigma-Aldrich, St. Louis, MO, USA) was added to each well, including the negative and positive control wells. The plates were then incubated for 8 h at 37 °C. Absorbance readings were taken at 570 and 600 nm wavelengths using a Tecan Infinite M200 Pro microplate reader (from Tecan Trading AG, Männedorf, Switzerland). Cell quantification occurred at intervals of 1, 4, and 6 days. Triplicate samples were prepared for each condition.

After six days of cultivation, the samples underwent a two-step washing process with PBS, then fixation for 10 min using a 3.5% formaldehyde solution from Sigma-Aldrich. Post-fixation, the cells were permeabilized to enhance staining results via a solution of 1% BSA and 0.1% Triton X-100, diluted in PBS. The cell cytoskeleton was stained with ActinRed 555 from Thermo Scientific, Waltham, MA, USA, while the dermal fibroblasts’ nuclei were stained using Hoechst 33342, also from Thermo Scientific, Waltham, MA, USA, diluted 1:1000. Subsequently, all samples underwent analysis utilizing a Nikon Eclipse TI Fluorescence Microscope, Tokyo, Japan, in the DAPI and TRITC channels.

*Staphylococcus aureus* (*S. aureus*, ATCC 25923) and *Escherichia coli* (*E. coli*, ATCC 25922) underwent 24 h cultivation on nutrient agar. Following this, overnight culture suspensions were blended with nutrient broth medium, adjusting the microorganism in the initial concentration of bacterial cells 1 × 10^6^ CFU/mL (colony-forming units per milliliter).

The Ti13Nb13Zr alloy samples, including those untreated, treated with the PEO process, and coated with AgNPs at concentrations of 0.125, 0.25, and 0.50 g/L, were submerged in a bacterial suspension. They were then horizontally incubated with 2.0 mL of the bacterial suspension in static conditions in a 24-well plate at 37 °C for 2, 4, 6, and 8 h. Subsequently, the disks underwent three washes with sterile sodium chloride solution to eliminate non-adherent cells. An ultrasonic bath (EMAG Ultrasonic cleaner Emmi-20 HC, EMAG AG, Mörfelden-Walldorf, Germany) was then used to sonicate the samples in tubes containing 1.0 mL of sterile saline solution for 1 min to remove bacteria adhered to the specimen surfaces. Following this step, 10 μL aliquots of saline solution were inoculated onto nutrient agar plates using the streak plate technique to quantify the bacterial count after 24 h of incubation at 37 °C.

## 3. Results

**Figure 1:**
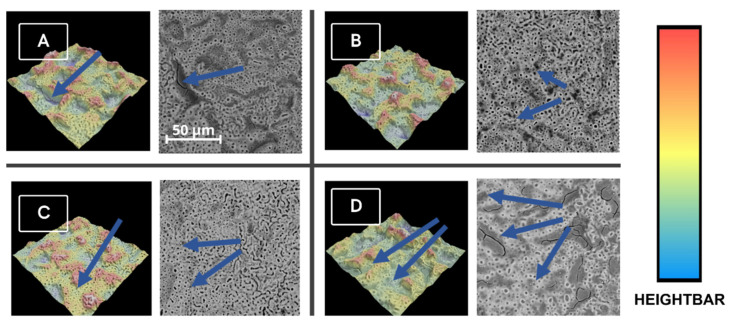
SEM images with corresponding 3D generated images of (**A**)—G sample; (**B**)—GS sample; (**C**)—GSE sample; (**D**)—GSE-A sample. Blue arrows point on the spots with the abrasive SiC material stuck on the surface of samples. The magnification of images is ×2000.

The following samples—G, GS, GSE, and GSE-A (250 V)—were investigated under an SEM microscope, and corresponding images of their surfaces were collected in Figure 1. In the G, GS, and GSE samples, abrasive grains remaining from the grinding and sandblasting processes are visible. GSE exhibits the propagation of the surface profile of the developed GS. The atrophy of grinding marks and the development of the surface in the GS samples are also evident in the GSE. The 250 V PEO-treated sample (GSE-A) surface exhibits a similar height profile and 3D structure to GSE, however, after the oxidation, the surface has been covered in regular, oval-shaped pits (Figure 1).

**Figure 2:**
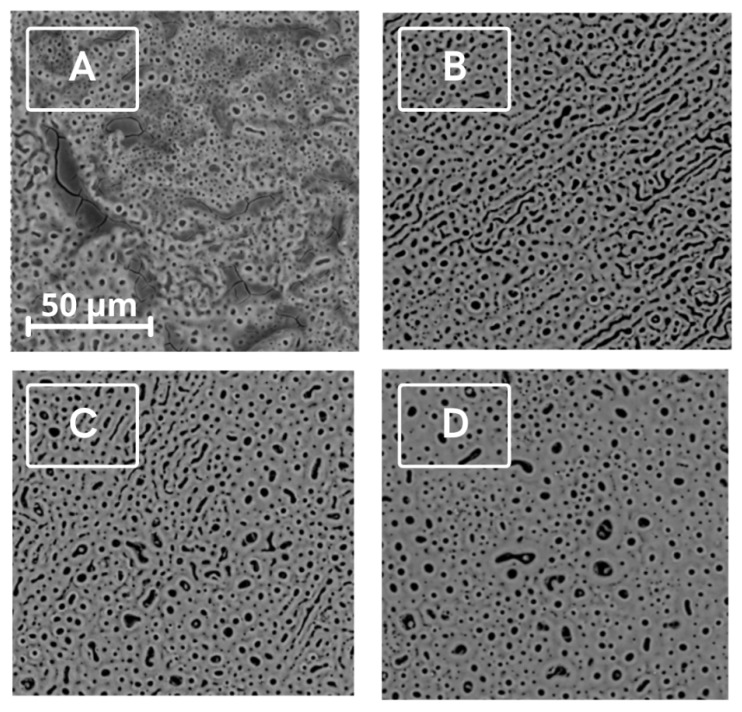
SEM images of the PEO-treated samples in 1M H_3_PO_4_: (**A**)—250 V; (**B**)—275 V; (**C**)—300 V; and (**D**)—325 V. The magnification of images is ×2000.

SEM images of the PEO-treated samples were taken, analyzed, and collected as in Figure 2. From these images, there is a visible trend, whereby with the increase in voltage, the size of obtained pores in the ceramic, oxide layer increases. As the voltage increases, more uniformly structured surfaces are being formed. The extent of the arrangement of the chaotic pores increases with the increase in voltage—for samples treated with 275 V, it is visible that small pores formed on the uniform surface are arranged in an array following grinding marks from the polishing step. A vague geometric pattern was found for samples oxidized in 300 V. The surface of the sample oxidized with 325 V had no geometric pattern of the pit formation and the size of the pores was the greatest of all investigated samples. The surface topography of samples anodized in 250 V was found to propagate the imperfections and random defects caused by the sandblasting process; therefore, it was concluded that this voltage would be applied to the rest of the samples.

**Figure 3:**
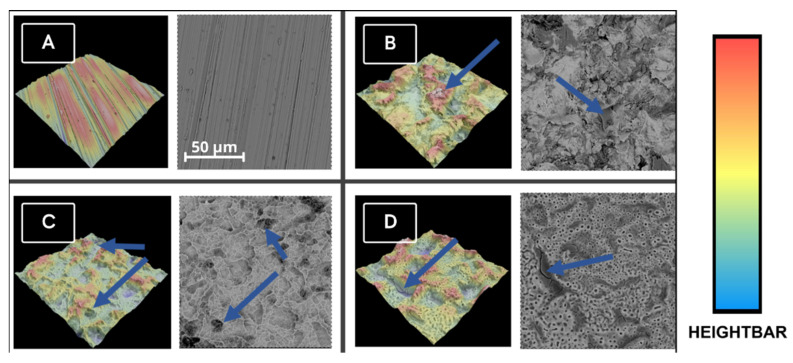
SEM images and according to 3D images of samples, where (**A**)—GSE-A sample with blue arrow showing cracked surface of the sample; (**B**)—TnZ GSEA Ag 0.125 sample; (**C**)—TnZ GSEA Ag 0.25; and (**D**)—TnZ GSEA Ag 0.5 sample. Blue arrows are pointing to the visible AgNP on (**B**–**D**).

Samples treated with a suspension of nanoparticles visually differ from the GSE-A sample (Figure 3). Visible small pores at the lowest points of the PEO sample disappear with the increasing concentration of the silver suspension. The quantity of silver nanoparticles and larger conglomerates visible on the surface of the samples increases with the increase in the concentration of silver nanoparticles. In the sample treated with a 0.5 g/L solution, complete sealing of the lowest localized pores of the coating is observed, which probably cracked during the sample drying process. In all samples, the pores of the structures visibly decrease in depth and size with the increasing concentration of the suspension, suggesting that more silver is deposited on the ceramic coating of the alloy.

Surface roughness measurements show that sandblasting greatly increases surface roughness compared to polished samples. According to data collected in Table 2, etching in oxalic acid did not affect the roughness of the surface significantly. However, PEO treatment in 1 M H_3_PO_4_ in 250 V increased the arithmetical mean value by 1.30 µm. The deposition of AgNPs on the surface of GSE-A did not affect surface roughness significantly.

**Figure 4:**
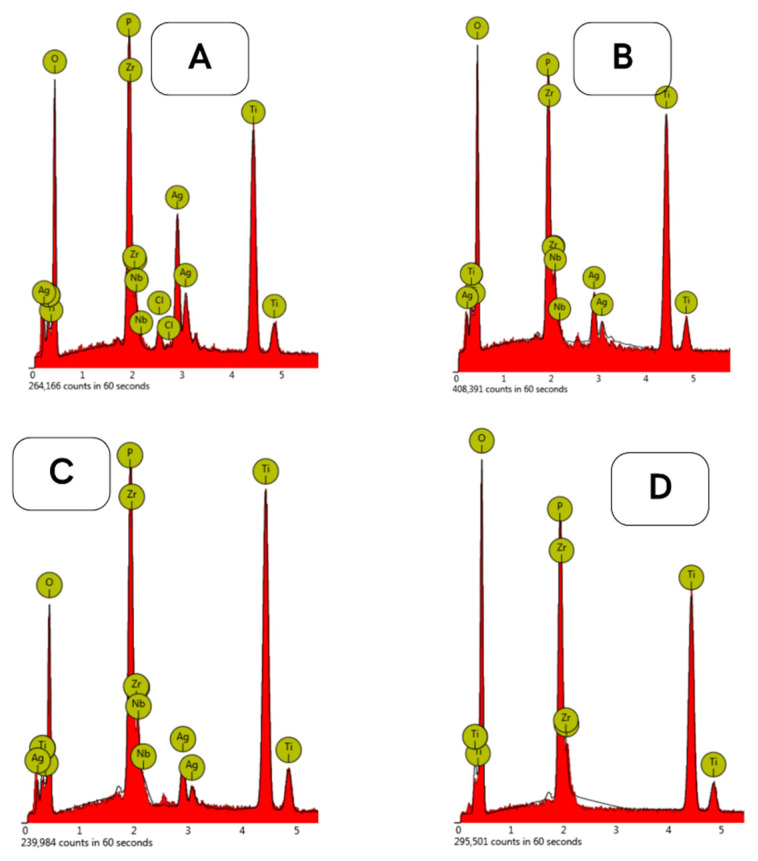
EDX analysis graphs of the samples where (**A**)—GSEA-0.5Ag sample; (**B**)—GSEA-0.25 Ag sample; (**C**)—GSEA-0.125 Ag sample; and (**D**)—GSE-A sample.

The energy-dispersive X-ray spectroscopy (EDX) provided a qualitative analysis of elements present on the surface of the investigated samples. Obtained EDX spectra were gathered in Figure 4 below. Nanocoated samples treated with silver suspension had silver atoms detected, in addition to the elemental components of the alloy and the elements incorporated in the oxide layer. Data presented in Table 2 show that with the increase in AgNP concentration in the suspension, the amount of silver deposited on the surface increases. All the samples contain incorporated phosphorus and high oxygen content due to the PEO process.

Results presented in Table 3 show that the polished surface exhibits the highest hydrophilicity having the smallest contact angle. Water droplets spread on its surface almost instantly. The sandblasted surface exhibits the most hydrophobic properties since the obtained contact angle for it is the highest. Acid etching decreased the value of contact angle, possibly by smoothing the surface and increasing the size of cavities and pores, allowing water to flow into them. The PEO surface shows an increase in the contact angle. There is no clear dependence between the CA value and the concentration of the silver nanoparticle suspension used. The surface with a silver concentration of 0.5 g/L exhibits the highest hydrophobicity among the nanocoated samples, while the surface with a silver content of 0.125 g/L shows the lowest.

**Figure 5:**
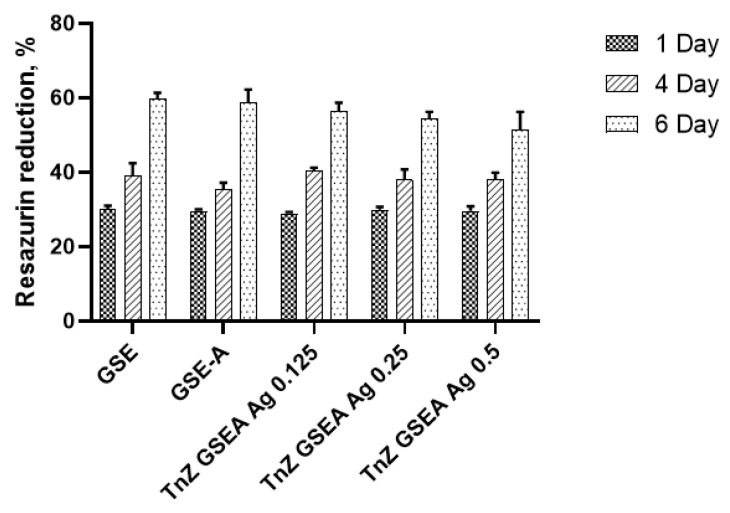
Resazurin reduction assay data on human dermal fibroblast proliferation during the 6-day experiment on different TnZ samples.

The data presented in Figure 5 indicate that the application of orthophosphoric acid to plasma-electrolyte-treated Ti13Nb13Zr alloy does not influence implant biocompatibility. At the same time, the novel coating did not influence fibroblast proliferation compared to the untreated surface. However, the addition of AgNPs at varying concentrations slightly influences biocompatibility, as compared to the untreated control samples. Notably, the lowest observed cytotoxicity correlates with the minimal AgNP concentration of 0.125 g/L. After a four-day incubation period, there was no significant difference in adhesion and proliferation among all sample groups.

**Figure 6:**
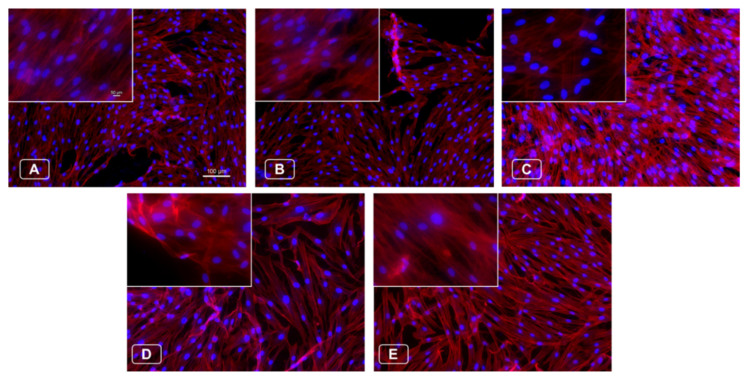
Fluorescent images of nuclei (blue) and cytoskeleton staining (red) on the 6th day of dermal fibroblasts on the samples, where (**A**)—TnZ G-S-E, (**B**)—TnZ G-S-E-A, (**C**)—TnZ G-S-E Ag 0.125 µg/mL, (**D**)—TnZ G-S-E-A Ag 0.25 µg/mL, (**E**)—TnZ G-S-E-A Ag 0.5 µg/mL. The magnification of the main images is ×100 (scale bar = 100 µm), and on the insets ×400 (scale bar = 50 µm).

Furthermore, upon a six-day incubation of the cells on the Ti13Nb13Zr alloy samples, fluorescence microscopy was employed to examine cell morphology. Here, the filamentous actin was highlighted with Rhodamine Phalloidin, emitting a red fluorescence, while the nuclei were counterstained with Hoechst, emitting a blue fluorescence, as showcased in Figure 6. The resulting images reveal an elongated cytoskeleton structure in the human dermal fibroblasts with oval-shaped nuclei, indicating typical morphology. A uniform distribution of fibroblasts was observed across both the control and nanoparticle-modified samples, suggesting that the surface modifications did not adversely affect the fibroblast distribution.

**Figure 7:**
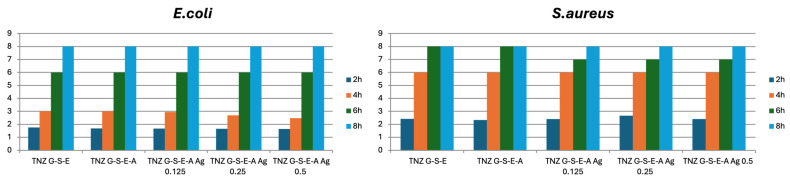
Time-dependent antibacterial effects on *E. coli* and *S. aureus* bacterial strain.

The initial seeded concentration of 10^6^ CFU/mL was used. There was a slight reduction in the *E. coli* population within the first 2–4 h of incubation, with a 2.9–4 Log10 CFU/mL decrease for samples containing 0.25 g/dm^3^ and 0.50 g/dm^3^. However, after 4–8 h of incubation, there was a concerning increase in the *E. coli* population, reaching up to 8 Log10 CFU/mL. This suggests that the AgNPs might lose effectiveness over time due to detachment from the alloy surface, reducing their availability to interact with bacteria (Figure 7).

The same effect was observed in the case of *S. aureus* (Figure 7).

## 4. Discussion

The presented research indicates that the proposed method of dropwise application of an AgNP suspension may prove to be a useful method of achieving bactericidal releasing-type coatings on the surfaces of PEO-H_3_PO_4_ implants. The acid-etched sample surfaces are similar in terms of topography, roughness, and wettability to the SLA surfaces of the titanium implants, which are proven to promote osseointegration and speed up the recovery period. It is clear that sandblasting and etching in oxalic acid developed and increased the surface area of samples. Scanning electron microscope images provide evidence that an AgNP suspension deposits onto the sample surface, as also supported by atomic composition studies using the EDX method. The conducted experiments demonstrate that plasma electrolytic oxidation is an effective tool for both preparing the sample surface through additional cleaning from abrasive materials and residues from etching and enabling the deposition of silver nanoparticles into the created pores. Plasma electrolytic oxidation of titanium and its alloys in acidic conditions forms a biocompatible layer of anatase, which is an α phase of titanium oxide. SEM images show that increasing the concentration of silver in the suspension increases the amount deposited on the sample surfaces. However, the use of a suspension with a concentration of 0.5 g/L resulted in flooding the lower layers of the surface and covering the pores. Contact angle analysis provides information about the wettability of samples, which is higher for samples with silver nanocoating compared to the PEO sample. Achieved coatings may be described as hydrophilic, and may have similar properties to anti-adhesive self-assembling polymer coatings in terms of bacteriostatic properties. Most hydrophobic properties were observed for samples just sandblasted and acid-etched, where PEO treatment effectively decreased the contact angle, indicating an increase in hydrophilicity.

Several studies demonstrated the antibacterial, anti-adhesive effects of nanosilver-doped titanium implants [75,76,77]. The mechanism of silver nanoparticle action involves the release of ions, leading to degradation of the peptidoglycan component of the cell wall, inhibition of protein synthesis, and interaction with DNA, ultimately resulting in bacterial cell death [78]. In our previous research, applied AgNPs exhibited a variable degree of effectiveness in disrupting the biofilms of all tested Gram-negative microbes, including *A. baumannii*, *P. aeruginosa*, *K. pneumonia*, and *Enterobacter* spp. [79]. Incorporating AgNPs into the PEO coating led to the release of silver during immersion tests, enhancing antibacterial properties and exhibiting activity against *S. aureus* through continuous Ag+ ion release [80]. It has been demonstrated that AgNPs incorporated into PEO coatings can decrease *S. aureus* bacterial cell amounts on the surfaces of Ti alloys at the 2 h and 4 h time points of the test [81]. However, Gram-positive bacteria tend to be more resistant to AgNPs due to differences in cell wall structure [82].

## 5. Summary

This study shows that PEO-treated Ti13Nb13Zr alloy offers a high degree of biocompatibility with human fibroblast cells. The surface treatment consisting of sandblasting, etching, and plasma electrolytic oxidation formed an oxide, ceramic layer that promotes the growth of human cells. On the other hand, the results suggest that while AgNPs exhibit promising initial bactericidal effects against *E. coli* and *S. aureus*, they may not be sustainable in the long term. Otherwise, dose-dependent efficacy was not admitted in applied concentrations of nanoparticles. In conclusion, our study underscores the necessity for additional research aimed at determining the optimal concentration of silver nanoparticles for the Plasma Electrolytic Oxidation (PEO) treatment of metal alloys. By refining this parameter, we can potentially enhance the efficacy and performance of PEO treatments, paving the way for improved antibacterial surface properties.

## Figures and Tables

**Table 1:** Explanation of labeling of samples and treatments that have been conducted.

Label	Polished	Sandblasted	Acid-Etched	PEO	Silver Suspension [g/L]		
					0.125	0.25	0.5
G	×						
GS	×	×					
GSE	×	×	×				
GSE-A	×	×	×	×			
GSEA Ag 0.125	×	×	×	×	×		
GSEA Ag 0.25	×	×	×	×		×	
GSEA Ag 0.25	×	×	×	×			×

**Table 2:** EDX analysis—average weight percentage composition [*w*/*w*%] of investigated samples and their standard deviations.

	0.5	0.25	0.125	PEO
Element Symbol	%Weight (Stand. Deviation) [*w*/*w*%]			
O	46.59 (2.54)	49.48 (4.23)	43.93 (2.24)	51.96 (1.92)
Ti	22.67 (1.26)	21.99 (2.42)	29.02 (5.62)	22.58 (2.53)
Nb	2.54 (0.34)	3.13 (0.54)	2.48 (0.12)	3.82 (0.68)
Zr	10.45 (0.92)	11.29 (1.45)	12.85 (1.12)	14.12 (2.55)
P	6.74 (1.02)	6.26 (0.74)	7.68 (1.23)	7.52 (0.41)
Ag	11.01 (1.67)	7.85 (1.21)	4.04 (0.22)	

**Table 3:** Average contact angle and roughness of the surface of the samples.

Label of Sample	Average Contact Angle [°]	Sa [µm]
G	26.3 ± 6.9	1.87 ± 0.13
GS	93.4 ± 3.7	3.18 ± 0.21
GSE	41.2 ± 4.1	3.31 ± 0.26
GSE-A	55.2 ± 2.2	4.61 ± 0.19
TnZ GSEA Ag 0.125	34.4 ± 3.0	4.51 ± 0.54
TnZ GSEA Ag 0.25	46.6 ± 4.9	4.51 ± 0.23
TnZ GSEA Ag 0.5	30.9 ± 3.3	4.39 ± 0.18

## Data Availability

Data are available on request.
